# An economic evaluation of the healthcare cost of tinnitus management in the UK

**DOI:** 10.1186/s12913-017-2527-2

**Published:** 2017-08-22

**Authors:** David Stockdale, Don McFerran, Peter Brazier, Clive Pritchard, Tony Kay, Christopher Dowrick, Derek J Hoare

**Affiliations:** 1 British Tinnitus Association, Ground Floor, Unit 5, Acorn Business Park, Woodseats Close, Sheffield, S8 0TB UK; 2 Colchester Hospital University NHS Foundation Trust, Lexden Rd, Colchester Essex, CO3 3NB UK; 3 Optimity Advisors, 1st Floor Kemp House, 152-160 City Rd, London, EC1V 2DW UK; 4 Wickenstones Ltd, Unit 26, 127 Olympic Avenue, Milton Park, OX14 4SA UK; 5 Aintree University Hospital NHS Foundation Trust, Lower Lane, Liverpool, L9 7AL UK; 6 Department of Psychological Sciences, University of Liverpool, Liverpool, L69 3GL UK; 7 NIHR Nottingham Biomedical Research Centre, Otology and Hearing Group, Division of Clinical Neuroscience, University of Nottingham, Nottingham, NG1 5DU UK

**Keywords:** Tinnitus, Hearing aids, Cost effectiveness, Ear nose and throat, General practice, Audiology, Clinical psychology, Cognitive behaviour therapy, Hearing aids

## Abstract

**Background:**

There is no standard treatment pathway for tinnitus patients in the UK. Possible therapies include education and reassurance, cognitive behavioural therapies, modified tinnitus retraining therapy (education and sound enrichment), or amplification of external sound using hearing aids. However, the effectiveness of most therapies is somewhat controversial. As health services come under economic pressure to deploy resources more effectively there is an increasing need to demonstrate the value of tinnitus therapies, and how value may be continuously enhanced. The objective of this project was to map out existing clinical practice, estimate the NHS costs associated with the management approaches used, and obtain initial indicative estimates of cost-effectiveness.

**Methods:**

Current treatment pathways, costs and health outcomes were determined from the tinnitus literature, national statistics, a patient survey, and expert opinion. These were used to create an Excel-based economic model of therapy options for tinnitus patients. The probabilities associated with the likelihood of an individual patient receiving a particular combination of therapies was used to calculate the average cost of treatment per patient, average health outcome per patient measured in QALYs gained, and cost-effectiveness, measured by the average cost per QALY gained.

**Results:**

The average cost of tinnitus treatment per patient per year is GB£717, equating to an NHS healthcare bill of GB£750 million per year. Across all pathways, tinnitus therapy costs £10,600 per QALY gained. Results were relatively insensitive to restrictions on access to cognitive behaviour therapy, and a subsequent reliance on other therapies.

**Conclusions:**

NHS provisions for tinnitus are cost-effective against the National Institute for Health and Care Excellence cost-effective threshold. Most interventions help, but education alone offers very small QALY gains. The most cost-effective therapies in the model were delivered within audiology.

**Electronic supplementary material:**

The online version of this article (doi:10.1186/s12913-017-2527-2) contains supplementary material, which is available to authorized users.

## Background

Tinnitus is the phantom sensation of sound, often a ringing, hissing, or buzzing that is experienced by about 10% of the population. It is often medically unexplained (subjective), chronic, and for some people can significantly impair quality of life [1–3]. Clinical management for the most part relies on counselling or cognitive therapy [4, 5], managing any associated hearing difficulties with hearing aid amplification [6], or masking the tinnitus percept using sound devices such as hearing aids or sound generators [7, 8]. Most tinnitus management options are poorly researched and might be considered experimental or even controversial [9, 10].

The most commonly offered treatment modality is a combination of education, counselling, and sound therapy based on a neurophysiological model of tinnitus [11]. The clinical correlate of this model has been published as a formal management paradigm called Tinnitus Retraining Therapy (TRT) [12]. TRT *per protocol* is resource intensive and is not generally funded within the UK National Health Service (NHS). A pared down version of TRT is, however, widely used and is referred to as modified TRT (MTRT; [13]) in the current study.

Although a best practice commissioning guide for primary, secondary, and tertiary services for tinnitus has been published by the Department of Health [14], services across the UK vary enormously in terms of the patient pathway and the treatments that are offered or available. Audiology services for tinnitus may be accessible either directly via the GP, or only indirectly via GP referral to ENT [15]. By either route, referral rates differ dramatically across GPs [16]. Only two thirds of services offer some form of cognitive behavioural therapy (CBT), and clinical psychologists are rarely accessible [17].

In the developed world, tinnitus can be associated with significant healthcare costs. A retrospective US study by Goldstein et al. [18] estimated the healthcare cost of tinnitus to be around US$660 per patient per year, while in the Netherlands Maes et al. [19] have estimated the mean annual tinnitus-related healthcare cost per patient to be €1544.

In the UK, the incidence of bothersome tinnitus presenting to the NHS is increasing [20], giving rise to increasing costs. There is a need to examine the costs of tinnitus care in the UK, and provide a benchmark for the economic evaluation of new therapies or pathway redesign studies.

## Methods

### Study design

The study involved a collaborative effort between the British Tinnitus Association (DS), Optimity Advisors (PB, CP), and an advisory group comprising members with expertise in tinnitus, from backgrounds in audiology (TK, Beth-Anne Culhane, Peter Byrom), ENT (DM), General Practice (CD), and research (DJH). It is reported according to the consolidated health economic evaluation reporting standards (CHEERS; [21]).

We mapped out the clinical pathways and treatment options used in people presenting to their GP with symptoms of tinnitus. Costs and probabilities of a patient receiving a particular treatment were attached to the various treatments along the different clinical pathways in order to generate an overall average NHS treatment cost. Clinical pathways were defined based on expert clinical opinion. The intention was to produce a framework which broadly describes existing treatment patterns in the NHS for tinnitus patients. The model developed therefore does not compare different treatment options for a population with given characteristics who have tinnitus. Rather, it provides a baseline for the overall treatment costs and health outcomes for the generality of patients with tinnitus, given a set of assumptions about the likelihood of being managed in various ways.

An Excel model was constructed mapping out the most common treatment pathways (involving education and reassurance, discharge and self-management, hearing aids, CBT, MTRT, clinical psychology) and a cohort of patients run through the model. The range of treatment options in the model is not exhaustive of all possibilities as there may be atypical local models, or patients may leave a tinnitus pathway to enter a mainstream mental health pathway or a non-NHS healthcare service. Variation in the severity of tinnitus was not explicitly incorporated into the model. The only feature distinguishing patients in terms of clinical presentation was candidacy for hearing aids which is determined only after referral from the GP for further assessment; note practices in the prescription of hearing aids for tinnitus management are highly variable, particularly where there is milder or higher frequency hearing loss, and are very much influenced by the clinical experience and opinion of individual audiologists [6]. Drawing on previous modelling work, tinnitus patients either did or did not experience an improvement in their condition. Those who experienced an improvement either successfully habituated (were ‘cured’) or did not successfully habituate. Those who did not successfully habituate were discharged to self-management. Those whose tinnitus does not improve can be referred on for further treatment.

**Fig. 1:**
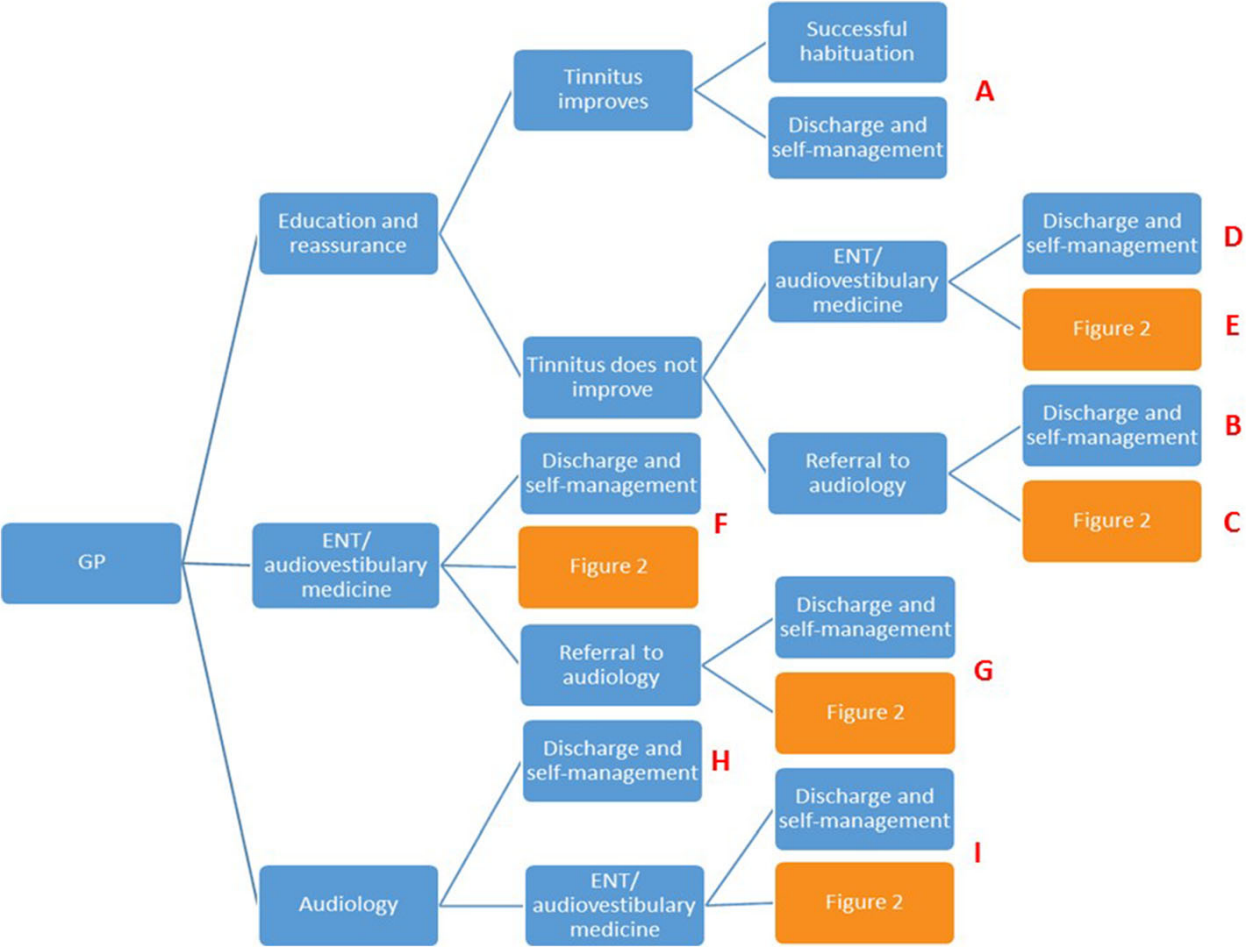
Clinical pathways. Initial presentation in the model starts with a General Practitioner (GP) consultation progressing to successive levels of onward referral, treatment, and ending with successful habituation (health benefit) or discharge to self-manage (no health benefit). ENT = ear nose and throat surgeon. Follow on treatment pathways are given in Fig. 2. Pathways are identified by letter (red text) and correspond to those in Table 1

**Fig. 2:**
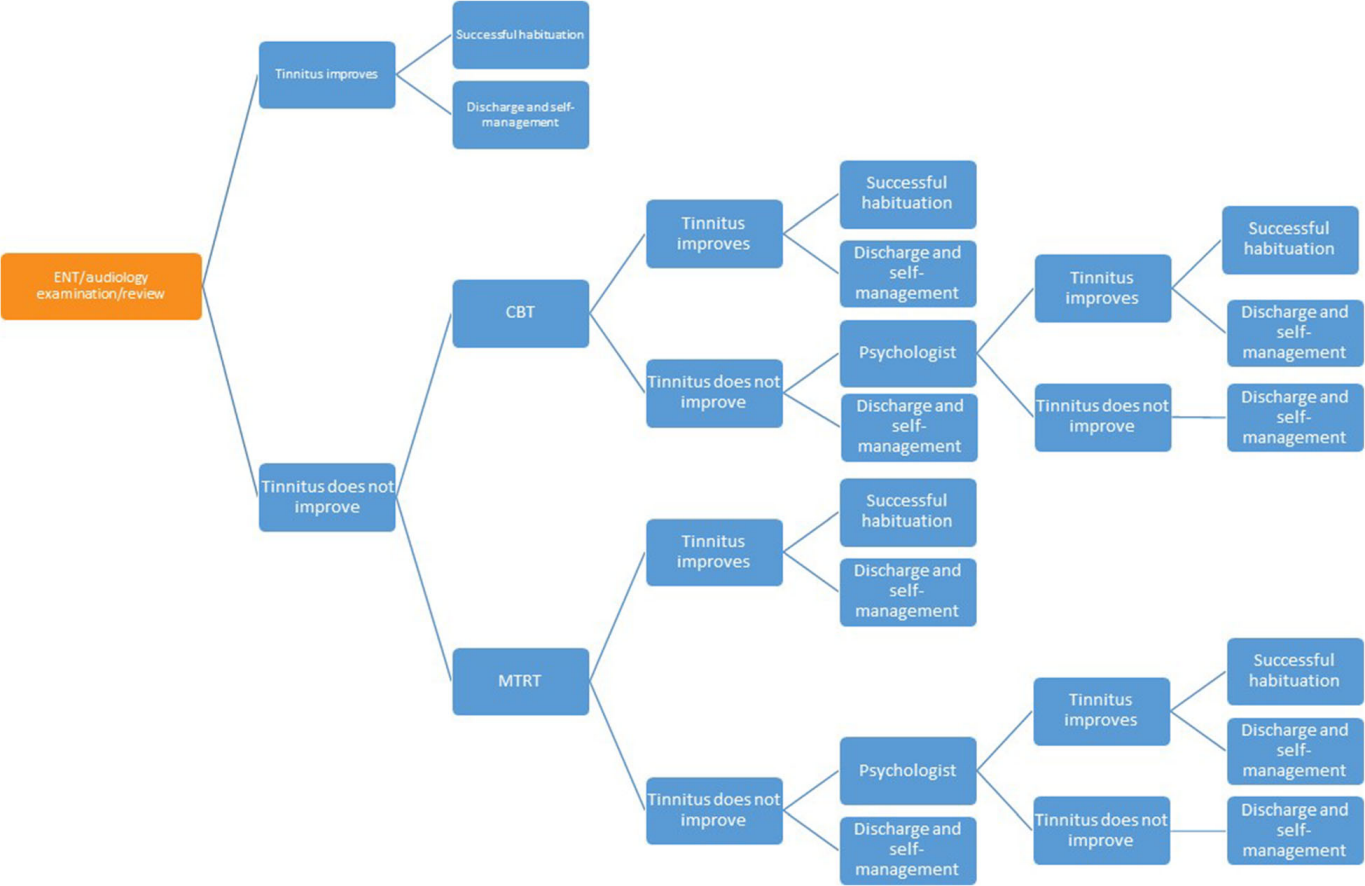
Follow-on treatment pathways. Following on from ENT/Audiology examination and review tinnitus patients progress to successive levels of onward referral, treatment, and ending with successful habituation (health benefit) or discharge to self-manage (no health benefit). CBT = cognitive behaviour therapy; ENT = ear nose and throat surgeon; MTRT = modified tinnitus retraining therapy. For those considered candidates for hearing aids the treatment options corresponding to MTRT and CBT are education and reassurance, hearing aids and maintenance, CBT plus hearing aids and maintenance, and MTRT plus hearing aids and maintenance

Of the cohort of patients presenting to a GP for the first time, an estimated 30% are referred to ENT, 7% are referred to audiology and 63% receive education and reassurance from the GP and are not referred onwards [16]. Patients who are unsuccessful in habituating to their condition after seeing their GP are subsequently referred to ENT or audiology. Figures 1 and 2 present illustrative pathways captured by the model. Those undergoing evaluation by an audiologist or Ear Nose and Throat (ENT) specialist can be referred on for further treatment with modified tinnitus retraining therapy (MTRT) or cognitive behavioural therapy (CBT). For simplicity, Fig. 2 illustrates the treatment options for those not considered candidates for hearing aids. For those considered candidates for hearing aids on audiological/ENT assessment, the treatment options corresponding to MTRT and CBT are education and reassurance, hearing aids and maintenance, CBT plus hearing aids and maintenance and MTRT plus hearing aids and maintenance. We acknowledge that it may not be appropriate to allocate the entire cost of hearing aids to the treatment of tinnitus. However, given the difficulty of estimating the proportion attributable and in order not to bias the analysis in favour of treatment for tinnitus, we included the full cost of hearing aids, and conducted a sensitivity analysis to test the effect of this assumption.

It is assumed that the time from initial GP consultation to eventual successful habituation or discharge will be less than 12 months. Hence new treatment interventions will have all been exploited in the first year. Recurring costs are those associated with ongoing support. For example, the costs of hearing aid maintenance will be incurred over the lifetime of the patient. Annual costs were discounted at a rate of 3.5% per annum as used by NICE for health technology appraisals. The result is the present value of the lifetime cost for a patient following each of the defined clinical pathway options.

### Sources of probabilities

The analysis draws on a number of sources of probabilities (Additional file 1). These include key journal publications, the results of a yet unpublished survey of the experiences of members of British Tinnitus Association (BTA), national statistics, and the clinical expert knowledge of the study team. Expert opinion was used to formulate a consensus, through discussion, on the current common clinical pathways followed by tinnitus patients.

Research evidence was used to estimate the following parameter values: the probability that tinnitus improves after CBT [22], the probability of receiving a hearing aid prescription [23], the probability that tinnitus improves after MTRT/CCBT and hearing aids [24], the probability of receiving a hearing aid [25], the probability of onward referral by GP and reattendance [16], the probability of improvement after hearing aid fitting or education and reassurance [26], the probability of receiving an audiology intervention, [15, 27, 28], CBT effectiveness [29], the probability of receiving MTRT [30], and the probability that tinnitus improves after CBT and hearing aids [31]. Member survey data were used to estimate values for another group of variables in the model such as the proportion of patients discharged rather than having onward referral by ENT. Remaining transition probabilities were obtained from a survey of experts, with disagreement (and logical inconsistencies) resolved by consensus.

### Comparator

The implicit comparator was a patient who did not seek treatment for their condition and was assumed to incur zero tinnitus-related NHS treatment costs. In addition to treatment costs, a quality adjusted life year (QALY) gain was attributed to those patients who successfully habituated compared with those who are discharged at any stage and left to self-manage and relative to those who do not seek treatment. In the absence of good natural history data at the time of analysis, a hypothetical zero cost, zero QALY cohort at least provides a common comparator.

### Modelling common tinnitus pathways

#### Probabilities

Table 1 gives illustrative examples of numbers following given pathways and the numbers of those habituating to their condition. Further description of these pathways is given in Additional file 2.

**Table 1:** Probabilities of overall distribution of 100 tinnitus patients to pre-defined patient pathways, successful habituation, and discharge to self-management

	Route	Total people	Total number of successful habituation	Total number of people discharged to self-manage
A	GP to Education and reassurance	23.31	13.29	10.02
B	GP to Education and reassurance to Audiology	14.40	4.28	10.11
C	GP to Education and reassurance to Audiology to ENT/audiovestibular medicine	1.08	0.01	1.07
D	GP to Education and reassurance to ENT/audiovestibular medicine	16.95	0.17	16.78
E	GP to Education and reassurance to ENT to Referral to audiology	7.26	2.16	5.10
F	GP to ENT/audiovestibular medicine	21.00	0.21	20.79
G	GP to ENT/audiovestibular medicine to Follow-on audiology	9.00	2.68	6.32
H	GP to Audiology	6.51	1.936	4.574
I	GP to Audiology to ENT/audiovestibular medicine	0.4900	0.0053	0.4847

#### Costs

The direct NHS healthcare costs were captured or estimated for each clinical pathway including the costs of clinical consultations (GP, ENT, audiologist/hearing therapist, clinical psychologist), diagnostic assessments, and management options including sound devices (hearing aids, sound generators), pharmacotherapy, CBT, and MTRT. Unit costs and source references used in the model are given in Table 2 and Additional file 3. A number of assumptions related to how often costs are incurred were derived from expert knowledge and the BTA member survey. Examples of modelling assumptions are that:

- 50% of patients seen in audiology/ENT/audiovestibular medicine undergo an MRI scan;
- hearing aids are reassessed and replaced every 4 years;
- follow-up, repairs and maintenance of hearing aids are undertaken annually;
- replacement of packs of 6 hearing aid batteries occurs 6 times a year;
- one pair of hearing aids is issued to all patients using this management modality;
- patients receive on average two GP appointments before they are first referred onwards;
- patients treated by a Clinical Psychologist are seen twice in the initial year.

**Table 2:** Annual tinnitus-related treatment costs

Treatment	Yearly cost
Digital Hearing Aids	£85.00^a^
Hearing aids assessments	£65.00^a^
Hearing aids fitting	£65.00^a^
Hearing aids follow up	£108.00^a^
Hearing aid batteries	£12.00^b^
Hearing Aid Repairs	£52.00^a^
Cognitive Based Therapy (CBT) (all associated staff, diagnostic and operating cost and consist of 3 sessions per year)	£471.00^a^
Tinnitus Therapy plus wearable sound generator (3 sessions with mid-point band level 6 for an allied health care professional)	£303.00^c^
GP session (11.7 min)	£52.00^c^
Pharmacotherapy - betahistine	£25.12^d^
Pharmacotherapy - Amitriptyline	£13.29^e^
Magnetic Resonance Imaging (MRI)	£85.00^a^
Associate Medical Specialist in ENT/Audiovestibular medicine (1 h)	£121.00^c^
Audiologist (1 h)	£18.76^e^
Clinical Psychologist (2 h)	£268.00^c^

^a^NHS trust reference cost schedules 2012–2013 [41] https://www.gov.uk/government/publications/nhs-reference-costs-2012-to-2013

^b^ www.hearing-aid-batteries.org.uk [42]

^c^Unit costs of Health and Social Care 2013.http://www.pssru.ac.uk/project-pages/unit-costs/2013/ [43]

^d^National Drug tariff https://www.nhsbsa.nhs.uk/nhs-prescription-services [44]

^e^expert opinion

The total annual healthcare cost of tinnitus in the NHS was calculated by multiplying the average cost per pathway by the proportion of patients per pathway in the model, and the estimated annual number of patients seeking NHS care for tinnitus in the UK.

#### Health outcomes

Health outcomes from a patient perspective were expressed in terms of quality adjusted life years (QALYs) per person where QALYs gained represent the number of years in good health which can be expected over the individual’s remaining lifetime (assumed to be 35 years) under NHS treatment relative to the comparator of no NHS treatment. Patients who successfully habituate to tinnitus gain QALYs, whereas patients who are discharged without treatment and self-manage in an undirected way have no QALY gain. An annual QALY gain of +0.02 (discounted QALY over 35 years = 0.4) was applied for all patients who successfully habituated to their tinnitus. No directly relevant studies on quality of life improvement suitable for calculating QALYs were found. However, an average ear disease pre-post management score on the Health Utilities Index mark 3 (Hui-3 [32]) has been reported by Swan et al. [33]. It was assumed that the pre-treatment quality of life applies over the lifetime for those who do not seek NHS care. The annual QALY gains in future years were discounted at a rate of 3.5% to determine a present value of lifetime health benefit.

#### Cost-effectiveness

Cost-effectiveness results were obtained by multiplying the relevant costs, QALYs and probabilities along each pathway considered. The assumptions underlying the cost-effectiveness results need to be borne in mind when interpreting the results presented below. As the main purpose of the study was to cost the range of possible pathways for the management of tinnitus, the cost-effectiveness results should be regarded as illustrative. We acknowledge the limitations of the analysis and highlight areas for further research in a later section.

## Results

### Cost effectiveness of treating tinnitus

Costs and health outcomes are not reported here for all the pathways identified in Figs. 1 and 2. Costs, QALYs, and cost per QALY gained (relative to no treatment) are reported for those who habituate to their condition after receiving education and reassurance only, and for those whose condition does not improve and who are referred to, or who are referred directly by their GP, to:

- Audiology
- Ear Nose and Throat (ENT)/audiovestibulary medicine review
- Audiology followed by ENT review
- ENT review followed by audiology

This gives eight additional pathways: those referred directly to one of these four and those whose condition does not improve following education and reassurance by the GP. The results for each pathway summarise the costs and QALYs accrued by patients over the course of their remaining treatments, potentially up to consultation with a clinical psychologist. The results are therefore averages for those who successfully habituate and those who do not successfully habituate at the end of their treatment journey. These ‘root’ pathways include those considered candidates for hearing aids (following ENT or audiology examination) as well as those who were not considered candidates.

Table 3 shows the costs and QALY gains for these pathways. Results for the first treatment possibility, education and reassurance from the GP, are reported for those whose tinnitus improves and who habituate to their condition or are discharged to self-management. The other pathways relate to those whose condition does not improve after GP education and reassurance and are referred to audiology/ENT or are referred directly to audiology/ENT. Across all pathways, tinnitus therapy costs GB£10,600 per QALY gained and would be regarded as cost-effective against the NICE benchmark of GB£20,000 per QALY gained.

**Table 3:** Costs and outcomes for key tinnitus management pathways

Pathway	Average Cost per person	Average QALY gain per person	Cost per QALY
GP to education and reassurance - tinnitus improves	£59	0.23	£258
GP to Educational reassurance to Audiologist	£2378	0.12	£19,988
GP to Educational reassurance to Audiologist to ENT	£354	0.004	£82,523
GP to Educational reassurance to ENT	£335	0.004	£83,250
GP to Educational reassurance to ENT to Audiologist	£2504	0.12	£21,015
GP to ENT	£335	0.004	£83,250
GP to ENT to Audiology	£2504	0.12	£21,015
GP to Audiology	2378	0.12	£19,988
GP to Audiology to ENT	£354	0.004	£82,523
Average across all pathways	£1051	0.10	£10,616

In the model the proportion of patients discharged directly from ENT is significantly higher than the proportion discharged from audiology. Of the small number of patients who are referred to ENT after seeing an audiologist, 97% of patients are discharged and self-manage after a full diagnostic review. In practice, patients who are referred to audiology are more likely to receive an intervention such as CBT, MTRT, with only 10% estimated to be discharged to self-management. Thus, the model generates a much higher cost per QALY for those pathways in which access to MTRT/CBT is mediated by ENT. Caution should be used when interpreting the results as they do not represent competing options for patients with given characteristics but are intended to capture the way in which patients (of all types) are managed in practice. Therefore, there may be clinical grounds for some patients being eligible for certain pathways and not others. The extreme cost-effectiveness ratio is due to the impact created from the number of patients who are discharged from the model at an early stage, receiving no treatment and therefore in our model having no improvement in health outcome.

### Sensitivity analyses

The sensitivity of the results to changes in the assumptions underlying the particularly high cost-effectiveness ratios associated with the ENT pathways was explored. In an early iteration of the model, the proportion of patients discharged rather than having onward referral by ENT following GP education and reassurance was estimated by the advisory panel to be just 15%. This would have given a cost effectiveness ratio of £7043/QALY for the GP to educational reassurance to ENT pathway. In comparison, the final model was revised using data from the members’ survey which placed the estimated discharge rate at 68% of those referred directly to ENT, and 97% of those referred from audiology to ENT. This is an important disparity between member report and clinician opinion that warrants further investigation.

A simulation was run to explore the effect of only allocating a proportion of the cost of hearing aids to tinnitus benefit. Hearing aids are often prescribed in the presence of tinnitus, but always with associated hearing loss, so some of the cost might reasonably be associated with improvements in listening abilities. In a sensitivity analysis where only 50% of the cost of hearing aids was attributed to the treatment of tinnitus the overall cost-effectiveness ratio was slightly reduced, to £9901/QALY.

A further simulation was run to examine the effect that varying the estimate of successful habituation would have on cost effectiveness ratios. In a simulation where only 20% of patients successfully habituate and 80% are discharged to self-manage after GP education and reassurance, the overall cost-effectiveness ratio increases to around £13,000/QALY. If only 15% of patients successfully habituated then the cost-effectiveness ratio would be around GB£18,600; if any fewer patients than 18% of patients at this point successfully improved then the cost effectiveness ratio was greater than the NICE GB£20,000 benchmark.

Further simulations were run to assess the effect of varying the QALY gain estimate. An estimated gain of 0.04 reduced the cost effectiveness ratio to less than GB£7000/QALY. If the gain was half that predicted, i.e. 0.01, then the cost effectiveness ratio would be around £15,000/QALY.

### Estimated total NHS healthcare cost of tinnitus

The average tinnitus-related healthcare cost per patient per year in the model was GB£717. Based on there being 43,900 GPs in the UK (nuffieldtrust.org.uk), each seeing two patients per month where the primary complaint is tinnitus [16] we estimate that 1.05 million GP consultations for tinnitus are conducted each year. As an estimate of total UK annual healthcare expenditure on tinnitus, this figure was multiplied by the average annual pathway cost per-patient (GB£717). This gives a UK healthcare cost of tinnitus in the region of GB£750 million which equates to about 0.6% of current annual public sector spending on healthcare in the UK [34]. If the relationship between healthcare and societal cost is proportionate to that reported by Maes et al. [19] then the societal cost of tinnitus to the UK is in the region of GB£2.7 billion per annum; however, empirical evidence is needed for a truer estimate.

## Discussion

This cost study benefited from an approach which synthesised different forms of evidence and expert knowledge to ensure the validity of the different approaches to tinnitus care evaluated. Overall, with a cost per QALY of GB£10,600, tinnitus therapy as currently delivered in the NHS is cost effective against the NICE benchmark of GB£20,000 per QALY gained. Cost effectiveness would be maintained if only 15% of patients successfully habituated to their tinnitus. The estimated cost-effectiveness is in line with those of Cima et al. [23] who trialled a specialised multi-disciplinary care package for tinnitus, estimating it to cost US$10,456–$24,580 per QALY gained [35]. Cost-effectiveness was found to be sensitive to the reported improvement in tinnitus (QALY gain) experienced by the patient, a variable on which there is limited information. Further clinical research quantifying the QALY improvements experienced by tinnitus patients would provide valuable additional insights to the cost-effectiveness of tinnitus therapies. Other assumptions in the model that are based on clinician opinion rather than data also limit confidence in the certainty of the estimates reported.

In the current model a small number of patients who successfully habituate after educational reassurance via their GP generate the smallest costs; a majority of patients are necessarily referred to ENT/audiovestibular medicine or audiology at their first or repeated GP consultation where they pick up further costs and benefits. Patients referred onwards by their GP were more likely to receive further treatment and successfully habituate to tinnitus as well as accruing greater costs. Patients referred through the audiology route were more likely to be offered a variety of interventions and thus more likely to successfully habituate than those not reaching audiology.

The UK NHS healthcare cost of tinnitus per person per year is estimated to be GB£717, which is comparable to estimates in other studies examining the costs of unexplained medical symptoms. Reed et al. [36] used patient data from the UK General Practice Research Database, estimating annual healthcare cost per patient with unexplained pain to vary between £582 and £925, depending on the severity of pain (judged by number of pain relief prescriptions over a three-month period). The figure for tinnitus falls substantially below the GB£1100 healthcare cost of tinnitus in the Netherlands estimated by Maes et al. [19] yet QALY estimates were comparable. However, the Netherlands operates a compulsory health insurance system and differences in unit cost are likely to explain some of this mismatch between estimates for the two countries. The Maes et al. [35] cost of hearing aids at US$1038 is about 10 times our estimate for an NHS hearing aid.

Annual UK NHS healthcare cost and societal costs were approximated here at £750 million, and £2.7 billion respectively. It is striking that the prevalence of tinnitus and likely cost to society are not reflected in the amount of funding to support tinnitus research, in the UK or elsewhere [37]. The UK healthcare and societal estimates would seem surprisingly low compared to those reported by Maes et al. [19]. They estimated that in the Netherlands the annual healthcare cost of tinnitus is €1.9 billion (2.3% of the country’s total healthcare expenditure) and societal cost to be €6.8 billion per year. Given the difference in population between the UK (60 m) and the Netherlands (12.5 m), this would imply an order of magnitude in the difference between the two estimates. However, Maes et al. [19] based their estimate on general tinnitus prevalence figures as opposed to patient numbers, i.e. assuming 10% (± 5%) of the population take up healthcare services for tinnitus. The true figure is likely to be much lower than even the lower estimate in their sensitivity analysis.

Pathways ending at ENT did not demonstrate cost-effectiveness, primarily due to an unexpectedly high rate of patients being discharged to self-manage, which in the model was associated with no QALY gain. In contrast only 10% of audiology patients were estimated to be discharged to self-manage. These figures had a very significant effect on estimates of cost-effectiveness and although based on the best available evidence, need confirmation or correction through future service evaluations. ENT is an essential element of the patient pathway for many tinnitus patients and as with any medically unexplained condition there is significant expenditure associated with investigations seeking disease [38]. Appropriate referral is therefore particularly important to cost effectiveness. For example, patients with tinnitus are unlikely to benefit from repeated referrals to ENT services which have already investigated any other likely diagnoses.

New pathways should explore ways of reducing unnecessary healthcare costs and at the same time improve quality of care for those with tinnitus. One model recently published is a ‘one-stop tinnitus service’ [39] where the tinnitus patient is assessed by both an audiologist and an ENT surgeon within the same appointment. In this model the patient has a full diagnostic work-up including routine imaging, and leaves with a management plan, which may be organised through their local audiology services. Whilst the authors aim to make care accessible and reduce unnecessary follow-up appointments, they acknowledge that the success of the model is dependent on appropriate referral. Until alternative tinnitus care models such as this are fully evaluated, they remain controversial. The recent American Academy of Otolaryngology (AAO) clinical practice guidelines [40] explicitly recommend against the routine use of imaging as appears to be used by Farr et al. [39]. These authors argue that it is a useful form of reassurance to patients, promotes acceptance of their state, and helps reduce anxiety, whereas the AAO guidelines conclude a preponderance of harm over benefit.

## Conclusions

Overall, the cost-effectiveness of current UK tinnitus therapies of £10,600 per QALY gained compares favourably with the generally accepted GB£20,000/QALY threshold. Further clinical research quantifying the QALY improvements experienced by tinnitus patients would support precise evaluations on the clinical and cost effectiveness of alternative models of patient triage and management, alternative patient pathways, and novel management options. Future studies could also test our estimate of societal costs directly with a large UK population to quantify personal and loss of productivity costs. This will be particularly desirable should tinnitus pathways be redesigned or when new interventions are introduced. Whilst healthcare costs of two interventions may be equal, personal or loss of productivity costs may still differ substantially. Cima et al. [23] reported higher productivity costs, specifically days of work lost, for those patients receiving specialised care instead of usual care. Proposed predictors of healthcare and societal costs of tinnitus (tinnitus severity and duration, severity of depression, and age) should also be tested in a UK population.

## Additional files

Additional file 1: Probabilities. Probability values and sources corresponding to each transition in the model. (XLSX 66 kb)

Additional file 2: Tinnitus Management Pathway Definition. Pathway descriptions. (DOCX 15 kb)

Additional file 3: The model. Utilisation of each resource in the model. (XLSX 81 kb)

## Abbreviations

BTA: British Tinnitus Association
CBT: Cognitive behavior therapy
ENT: Ear nose and throat
GP: General practitioner
ICER: Incremental cost per QALY gained
MRI: Magnetic resonance imaging
MTRT: Modified tinnitus retraining therapy
NHS: National health service
NICE: National institute for health and clinical excellence
QALY: Quality adjusted life years
TRT: Tinnitus retraining therapy
