# Supplementary material for: An economic evaluation of the healthcare cost of tinnitus management in the UK
Source: BMC Health Serv Res. 2017 Aug 22;17:577. doi: 10.1186/s12913-017-2527-2 (PMC5567641; doi:10.1186/s12913-017-2527-2)
Supplement: Supplementary file 2 — Tinnitus Management Pathway Definition. Pathway descriptions. (DOCX 15 kb) [file 12913_2017_2527_MOESM2_ESM.docx]

Additional file 2.

| **Tinnitus Management Pathway Definition** |
| --- |
| GP to education and reassurance – This cohort of patients receives educational reassurance from their GP before successfully habituating or discharge and self-managing. No onward referral occurs for this cohort of patients |
| GP to education and reassurance to Audiology – All patients in this cohort receive educational reassurance and are referred to Audiology as there has not been an improvement in their tinnitus. Patients are subsequently discharged after audiology or enter a hearing loss or no hearing loss pathway |
| GP to education and reassurance to Audiology to ENT/audiovestibular medicine – All patients in this cohort receive education and reassurance and are referred to Audiology as there has not been an improvement in their tinnitus. The cohort of patients in this pathway are then referred onto ENT/audiovestibular medicine. After a full diagnostic review patients are either discharged or referred for further follow-up treatment |
| GP to education and reassurance to ENT/audiovestibular medicine - All patients in this cohort receive education and reassurance and are referred onto ENT/audiovestibular medicine. After a full diagnostic review patients are either discharged or referred for further follow-up treatment |
| GP to education and reassurance to ENT/audiovestibular medicine to Referral to audiology - All patients in this cohort receive education and reassurance and are referred to ENT/audiovestibular medicine as there has not been an improvement in their tinnitus. The cohort of patients in this pathway are then referred onto Audiology. After an audiology review patients can either be discharged or referred for further follow-up treatment |
| GP to ENT/audiovestibular medicine – This cohort of patients is initially seen by their GP before being referred onto ENT/audiovestibular medicine where they undergo a full diagnostic review before being discharged or referred for further follow-up treatment |
| GP to ENT/audiovestibular medicine to Referral to audiology – This cohort of patients is initially seen by their GP before being referred onto ENT/audiovestibular medicine where they undergo a full diagnostic review. The full cohort of patients in this pathway is then referred onto Audiology where they can be discharged or referred for further follow-up treatment |
| GP to Audiology - This cohort of patients is initially seen by their GP before being referred onto Audiology where they undergo a clinical examination from an audiologist and any clinical tests before being discharged or referred for further follow-up treatment |
| GP to Audiology to ENT/audiovestibular medicine - This cohort of patients is initially seen by their GP before being referred to Audiology where they undergo a clinical examination from an audiologist and any clinical tests. The full cohort of patients in this pathway is then subsequently referred to ENT/audiovestibular medicine where they undergo a full diagnostic review before being discharged or referred for further follow-up treatment |
